# Contrasting genetic patterns between two coexisting *Eleutherococcus* species in northern China

**DOI:** 10.1002/ece3.2118

**Published:** 2016-04-12

**Authors:** Sheng‐Hong Wang, Lei Bao, Tian‐Ming Wang, Hong‐Fang Wang, Jian‐Ping Ge

**Affiliations:** ^1^ State Key Laboratory of Earth Surface Processes and Resource Ecology and MOE Key Laboratory for Biodiversity Science and Ecological EngineeringCollege of Life SciencesBeijing Normal UniversityBeijing100875China

**Keywords:** East Asia, ecological niche modeling, *Eleutherococcus senticosus*, *Eleutherococcus sessiliflorus*, northern refugia, phylogeography

## Abstract

Climate oscillations are the key factors to understand the patterns in modern biodiversity. East Asia harbors the most diverse temperate flora, largely because an extensive terrestrial ice cap was absent during repeated Pleistocene glaciation–interglacial cycles. Comparing the demographic histories of species that are codistributed and are close relatives may provide insight into how the process of climate change influences species ranges. In this study, we compared the spatial genetic structure and demographic histories of two coexisting *Eleutherococcus* species, *Eleutherococcus senticosus* and *E. sessiliflorus*. Both species are distributed in northern China, regions that are generally considered to be sensitive to climatic fluctuations. These regions once hosted temperate forest, but this temperate forest was replaced by tundra and taiga forest during the Last Glacial Maximum (LGM), according to pollen records. Using three chloroplast DNA fragments, we assessed the genetic structure of 20 and 9 natural populations of *E. senticosus* and *E. sessiliflorus*, respectively. Extremely contrasting genetic patterns were found between the two species; *E. sessiliflorus* had little genetic variation, whereas *E. senticosus* had considerably higher levels of genetic variation (15 haplotypes). We speculated that a recent severe bottleneck may have resulted in the extremely low genetic diversity in *E. sessiliflorus*. In *E. senticosus*, populations in Northeast China (NEC) harbored all of the haplotypes found in this species and included private haplotypes. The populations in NEC had higher levels of genetic diversity than did those from North China (NC). Therefore, we suggest that both the NC and NEC regions can sustain LGM refugia and that lineage admixture from multiple refugia took place after the LGM elevated the local genetic diversity in NEC. In NEC, multiple genetic hot spots were found in the Changbai Mountains and the Xiaoxing'an Range, which implied that multiple locations in NEC may sustain LGM refugia, even in the Xiaoxing'an Range.

## Introduction

Comparative phylogeography of codistributed species seeks to understand general patterns in the evolutionary history of communities (Bermingham and Moritz 1998; Avise 2000). For example, comparative phylogeography studies have provided many insights on the response of temperate forests to climatic oscillation in North America and Europe (Hewitt 1999, 2000, 2001, 2004; Soltis et al. 2006). Many phylogeography studies have been taken in the Qinghai–Tibetan Plateau and subtropical region (Qiu et al. 2011; Liu et al. 2012), while few in temperate forest located in northern East Asia.

Using pollen record data, northern East Asia was most likely replaced with tundra and taiga forest during the Last Glacial Maximum (LGM, approximately 21,000–18,000 years ago), whereas temperate forests probably retreated to the South, below 30°N (Harrison et al. 2001; Qiu et al. 2011; Cao et al. 2015). Interestingly, molecular data have revealed that the genetic diversity of several species in northern East Asia is not low and that genetic divergence existed between populations in southern and northern East Asia. For example, a divergence in the chloroplast genetic diversity of *Juglans mandshurica* has been found in the southern and northern Yanshan Mountains (Bai et al. 2010). Similar patterns have been recovered in *Fraxinus mandschurica* (Hu et al. 2008), *Ostryopsis davidiana* (Tian et al. 2009), *Quercus mongolica* (Zeng et al. 2010, 2011), and *Acer mono* (Guo et al. 2014; Liu et al. 2014), although the locations of the divergence vary from case to case. All of these cases provide evidence that northern cryptic refugia probably existed widely throughout northern East Asia (Qiu et al. 2011). However, whether all species in northern East Asia were capable of surviving *in situ* during the LGM is unknown. Not enough cases have been taken in northern East Asia to gain a general dynamic picture of the temperate forest there (Qiu et al. 2011; Liu et al. 2012). Moreover, the majority of these studied species have been trees with distant phylogenetic relationships. What patterns should be expected for congeneric species occurring in the same range is unclear.

Codistributed congeneric species usually have similar life histories, mating systems, and overlapped species ranges, which may be expected a similar level of genetic diversity (Leffler et al. 2012). However, many of those codistributed congeneric species have typically diverged in their niche, probably due to competitive exclusion between closely related species (Gause et al. 1934; Cavender‐Bares and Pahlich 2009). These species may have selected different altitudes, soil conditions, mountain slopes, or other aspects (e.g., Maliouchenko et al. 2007). Hence, these codistributed close relatives may react differently to climate change. Comparisons of the demographic histories of these codistributed and closely related species may shed light on how climate change influences species ranges. For example, European white oaks have occasionally followed similar colonization routes during glacial cycles. Movements out of the Iberian and Italian Peninsulas have been clearly identified. The species also survived in separate refugia in the eastern Balkans by colonizing the West, which resulted in the reshuffling of haplotype distributions (Petit et al. 2002). As another example, four forest‐dependent bat species, endemic to Taiwan, differed in their tolerance to altitude, the lowland species have experienced genetic divergence due to the mountain barrier before the LGM, whereas high‐altitude specialists and altitudinal generalists have had greater levels of gene flow (Kuo et al. 2014).

**Figure 1:**
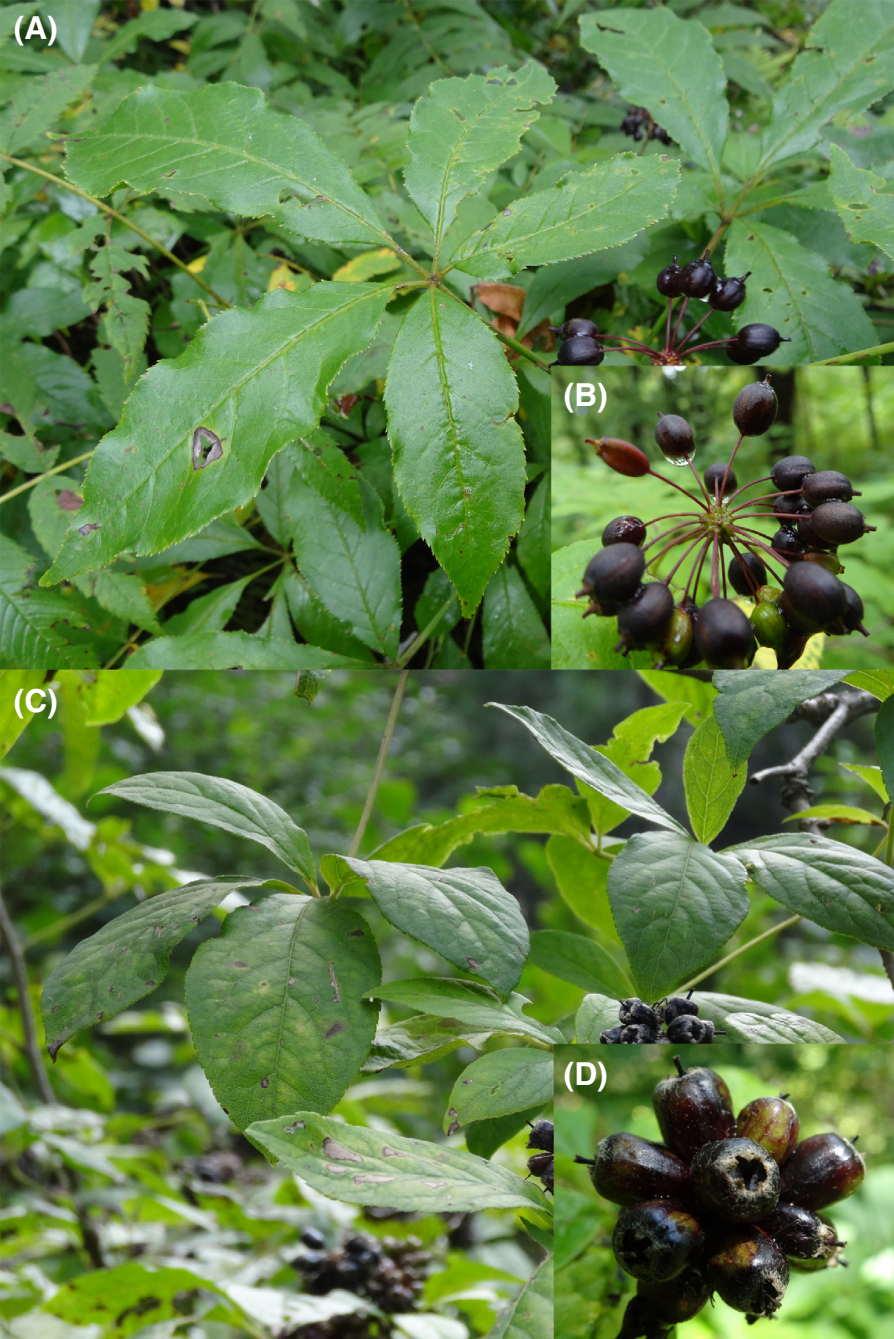
The individual plants of *Eleutherococcus senticosus* (A) and *E. sessiliflorus* (C), and their fruits (B) (D).

**Figure 2:**
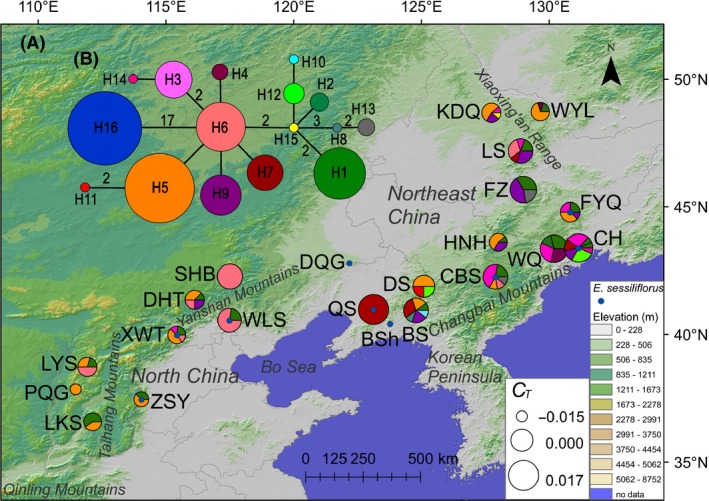
(A) Geographic distribution of 15 chloroplast DNA (cpDNA) haplotypes in *Eleutherococcus senticosus*. The size of the pies is proportional to the contribution of each population to the total gene diversity (*C*_T_). The blue dots represent the 9 sampling locations of *E. sessiliflorus*. (B) Median‐joining network of the 15 cpDNA haplotypes in *E. senticosus* and one cpDNA haplotype (H16) in *E. sessiliflorus*. Circle sizes correspond to haplotype frequency. Branch lengths longer than one mutational step are marked on each branch.

*Eleutherococcus senticosus* (Fig. 1A and B) and *E. sessiliflorus* (Fig. 1C and D) have similar ranges and biological characteristics. Both species are temperate deciduous shrubs that are primarily distributed in North China (NC) and Northeast China (NEC) within the Sino‐Japanese forest subkingdom (Fang et al. 2009) (Fig. 2A). Both species are pollinated by bees or butterflies (Liu et al. 2002a). *E. senticosus* is a trioecious species with bird‐ingested seed dispersal (Liu et al. 1997), whereas *E. sessiliflorus* is hermaphroditic and dichogamous (Liu et al. 2002b). Small differences also exist in the habitat selection of the two species. *E. senticosus* tends to be distributed at higher altitudes and latitudes. The altitude difference is notable in the southern part of the species range. In the southeastern area of Taihang Mountains, *E. senticosus* is generally found 300 m higher than *E. sessiliflorus*. This difference is generally consistent with descriptions from the Flora of China (Shang and Lowry 2007), because *E. sessiliflorus* is distributed at altitudes between 200 and 1000 m in China, whereas *E. senticosus* can reach 2000 m. This difference in altitude may reflect the differences in the sensitivity to temperature between the two species. Thus, whether these two species would respond differently to climate change is unknown. Because *E. sessiliflorus* appears to be more sensitive to temperature decreases, the range of *E. sessiliflorus* may shrink more severely than that of *E. senticosus*. *E. sessiliflorus* and *E. senticosus* are an ideal species pair to study the influence of climate change on the demographic history of species and on the level of genetic diversity. In this study, we used chloroplast DNA (cpDNA) sequence data and ecological niche modeling (ENM) to compare the demographic histories of the two shrub species. Three specific questions were asked: (1) Do the two codistributed species have similar levels of genetic diversity? (2) What are the differences in the population expansion patterns between the two codistributed species? (3) Where are the LGM refugia for the two species? Did these species occupy a single refugium or multiple refugia?

## Materials and Methods

### Population sampling

Leaf samples of 185 *E. senticosus* individuals from 20 natural populations and 61 *E. sessiliflorus* individuals from 9 natural populations were collected from 2012 to 2014 (Fig. 2A; Table 1). These sampled populations covered the ranges of both species. Within each population, a range of 4–15 individuals at least 30 m apart was sampled. Samples were dried and stored in silica gel until DNA was extracted.

**Table 1:** Sample locations, sample sizes, haplotypes, and genetic diversity of populations of *Eleutherococcus senticosus* and *E. sessiliflorus*

Population code	Location	Longitude (°E)	Latitude (°N)	Alt. (m)	*n*	*θ*	*π*	*Hd*	*R*_S_	Haplotype	Private haplotype
*Eleutherococcus senticosus*											
WYL	Wuyiling, Heilongjiang	129.65	48.73	425	10	0.00053	0.00052	0.511	1.067	H2 (2), H4 (1), H5 (7)	
KDQ	Kundeqi, Heilongjiang	127.74	48.68	440	9	0.00069	0.00060	0.694	1.603	H3 (1), H5 (5), H9 (2), H15 (1)	H15
LS	Liangshui, Heilongjiang	128.88	47.18	414	10	0.00080	0.00073	0.844	2.133	H1 (2), H3 (1),H6 (3), H7 (1), H9 (3)	
FZ	Fangzheng, Heilongjiang	128.99	45.67	297	9	0.00124	0.00154	0.722	1.563	H1 (3), H9 (4), H13 (2)	
FYQ	Fengyueqiao, Heilongjiang	130.82	44.79	728	8	0.00087	0.00094	0.821	2.000	H1 (2), H3 (2), H5 (3), H9 (1)	
HNH	Huangnihe, Jilin	128.00	43.61	650	9	0.00069	0.00065	0.639	1.317	H1 (1), H5 (5), H9 (3)	
CH	Chunhua, Jilin	131.15	43.39	690	15	0.00081	0.00100	0.867	2.269	H1 (1), H2 (1), H3 (4), H7 (2), H9 (2), H12 (4), H14 (1)	H14
WQ	Wangqing, Jilin	130.18	43.35	543	7	0.00077	0.00097	0.857	2.143	H1 (2), H2 (1), H3 (2), H4 (2)	
SHB	Saihanba, Hebei	117.50	42.27		8	0	0	0	0	H6 (8)	
CBS	Changbaishan, Jilin	127.89	42.25	1028	9	0.00138	0.00123	0.806	2.016	H1 (2), H3 (4), H5 (1), H6 (1), H13 (1)	
DS	Dasu, Liaoning	125.09	41.88	546	4	0.00123	0.00113	0.833	2.000	H5 (2), H11 (1), H12 (1)	H11
DHT	Dahaituo, Hebei	116.12	41.36	1611	8	0.00072	0.00064	0.821	2.000	H1 (1), H5 (3), H6 (2), H9 (2)	
QS	Qianshan, Liaoning	123.12	40.98	529	9	0	0	0	0	H7 (9)	
BS	Baishi, Liaoning	124.78	40.94	911	10	0.00146	0.00124	0.889	2.367	H1 (1), H5 (2), H7 (3), H8 (1), H9 (2), H10 (1)	H8, H10
WLS	Wulingshan, Hebei	117.48	40.56	1165	9	0.00041	0.00044	0.389	0.722	H1 (2), H6 (7)	
XWT	Xiaowutai, Hebei	115.42	39.98	1160	13	0.00061	0.00074	0.744	1.734	H1 (3), H3 (2), H5 (6), H6 (2)	
LYS	Luyashan, Shanxi	111.92	38.73	2060	10	0.00053	0.00058	0.689	1.476	H1 (2), H5 (3), H6 (5)	
PQG	Pangquangou, Shanxi	111.46	37.85	1944	10	0	0	0	0	H5 (10)	
ZSY	Zhangshiyan, Hebei	114.03	37.46	1537	8	0.00058	0.00080	0.536	0.929	H1 (3), H5 (5)	
LKS	Lingkongshan, Shanxi	112.13	36.58	1492	10	0.00053	0.00080	0.533	0.924	H1 (6), H5 (4)	
Average						0.00110	0.00086	0.835	2.413		
*Eleutherococcus sessiliflorus*											
FYQ	Fengyueqiao, Heilongjiang	130.82	44.79	478	1	–	–	–	–	H16	–
CH	Chunhua, Jilin	131.15	43.39	242	8	–	–	–	–	H16	–
DQG	Daqinggou, Neimenggu	122.17	42.8	211	3	–	–	–	–	H16	–
CBS	Changbaishan, Jilin	127.89	42.25	775	8	–	–	–	–	H16	–
QS	Qianshan, Liaoning	123.12	40.98	483	10	–	–	–	–	H16	–
WLS	Wulingshan, Hebei	117.48	40.56	899	11	–	–	–	–	H16	–
BSh	Baoshan, Liaoning	123.78	40.42	221	2	–	–	–	–	H16	–
XWT	Xiaowutai, Hebei	115.42	39.98	859	9	–	–	–	–	H16	–
ZSY	Zhangshiyan, Hebei	114.03	37.46	1221	9	–	–	–	–	H16	–
Average						–	–	–	–		

Sample size (*n*), Watterson's estimate (*θ*), nucleotide diversity (*π*), haplotype diversity (*Hd*) and allele richness after rarefaction (*R*_S_) are shown for each population.

### DNA extraction, amplification, and sequencing

Total genomic DNA was extracted using a plant genomic kit (Tiangen, Beijing, China). To check for polymorphisms, 13 commonly used cpDNA fragments were initially chosen for amplification in 8 individuals from 4 different populations (CH, CBS, WLS, and XWT, Fig. 2A), which represented the ranges of both species. These cpDNA fragments included *trnL‐trnF* (Taberlet et al. 1991), *trnD‐trnE*,* trnS* ^*UGA*^ *‐trnfM* ^*CAU*^ (Demesure et al. 1995), *rpl16* (Small et al. 1998), *atpB‐rbcL* (Hodges and Arnold 1994), *trnH‐psbA* (Sang et al. 1997; Tate and Simpson 2003), *trnS‐trnG* (Shaw et al. 2005), *ndhJ‐TabE* (Taberlet et al. 1991; Shaw et al. 2007), *psaI‐accD*,* rpl32‐trnL*,* atpI‐atpH*,* ndhA* (Shaw et al. 2007), and *matK* (Zhu et al. 2011). Polymorphisms were always detected in *E. senticosus* but not in *E. sessiliflorus*. Therefore, only 3 of the 13 cpDNA fragment sequences (*trnL‐trnF*,* rpl16*, and *matK*) were used for amplification in all sampled individuals of *E. senticosus* and *E. sessiliflorus*. Polymerase chain reaction (PCR) was performed in 40 μL, containing 10–20 ng of template DNA, 1 × buffer, 200 mmol/L each dNTP, 2.0 mmol/L MgCl_2_, 0.1 mmol/L each primer, and 2 units of *Taq* (TaKaRa, Dalian, China). Our PCR procedure comprised an initial denaturation at 95°C for 5 min, followed by 35 cycles of 1 min at 94°C, 50 sec at 54°C, and 1.5 min at 72°C, with a final extension at 72°C for 8 min. PCR products were sequenced at the Beijing Branch of Shanghai Majorbio Bio‐pharm Technology Co., Ltd. All of the cpDNA haplotypes were deposited in GenBank with Accession Nos. KU378054–KU378101.

### Genetic data analyses

Chloroplast DNA sequence data were edited and aligned using Codoncode Aligner 3.6.1 (Codon Code Corporation, http://www.codoncode.com/aligner/) with the Clustal module. Each indel was treated as a single site in all the following analysis. The genetic diversity parameters (*Hd*,* π*) (Nei 1987) and (*θ*) (Watterson 1975) of each population and each species were calculated in DnaSP 5.10.01 software (Rozas et al. 2003). A median‐joining network was constructed by NETWORK 4.6.1.1 (Bandelt et al. 1999). The spatial distribution of haplotypes was mapped in ArcMap 9.3 (ESRI, Inc., RedLands City, CA, USA). To account for differences in sample sizes, allele richness after rarefaction (*R*_S_) and the contributions to total gene diversity of each population (*C*_T_) were calculated with the method described by Petit et al. (1998) in Contrib 1.4 (https://www6.bordeaux-aquitaine.inra.fr/biogeco_eng/Scientific-Production/Computer-software/Contrib-Permut/Contrib). Rarefaction size was set to be the same as the smallest population (e.g., 4, as it was in DS in *E. senticosus*). The rarefaction analyses partitioned the contribution of *C*_T_ to *C*_S_ and *C*_D_, with *C*_S_ representing intrapopulation diversity and *C*_D_ representing interpopulation diversity. Population differentiation (*G*_ST_, *N*_ST_) was also calculated by Permut 1.0 (Pons and Petit 1996), with 1000 permutations. In addition, a Mantel test using GenAlEx 6.501 (Peakall and Smouse 2006) was performed to detect the relationship between the genetic and geographic distance of population. The genetic distance was calculated in MEGA 5.0 (Tamura et al. 2011).

Potential signals of selection in chloroplast sequences were tested by comparing the relative abundance of the nonsynonymous substitution rate (*d*_N_) and the synonymous substitution rate (*d*_S_) between sequence pairs of the *matK* gene from each haplotype in the two species. We used the Nei–Gojobori model (Nei and Gojobori 1986) in MEGA 5.0 (Tamura et al. 2011). *Z*‐tests were applied to determine whether *d*_N_ was equal to *d*_S_. When *d*_N_ equaled *d*_S_, we inferred that neutral sequence evolution had occurred. The statistic *Z* = (*d*_N_−*d*_S_)/SQRT(Var(*d*_S_) + Var(*d*_N_)) was computed, wherein the variance of the difference was calculated using the bootstrap method (500 replicates).

To assess the spatial genetic structure of populations, values of the fixation index $F_{C_{T}}$ were estimated by spatial analysis of molecular variance (SAMOVA) version 2.0 software (Dupanloup et al. 2002). The highest variance among groups ($F_{C_{T}}$) was calculated each time with a user‐defined group number (*K*). We obtained a series of $F_{C_{T}}$ values for group numbers (*K*) from 2 to 8. The optimal number of groups was determined by the highest $F_{C_{T}}$ value.

To infer the phylogenetic relationships between haplotypes in both species, we used Bayesian inference in Beast 1.8.0 (Drummond and Rambaut 2007). A constant‐size coalescent tree prior was used in the analysis with a TIM3+G substitution model determined by jModelTest 2.0.1 (Guindon and Gascuel 2003). A Markov Chain Monte Carlo (MCMC) chain length of 10^7^ was used and recorded 10^4^ trees. Because the two studied species shared no haplotypes, we treated *E. sessiliflorus* as an outgroup for the haplotype tree of *E. senticosus*. The convergence of MCMC simulations was determined by the ESS value in Tracer 1.5 (Rambaut and Drummond 2009). Calculations were not stopped until the ESS value was greater than 200, which was considered to be a sufficient mixture between parameters. The tree topology of the Bayesian tree was visualized in FigTree 1.4.0 (http://tree.bio.ed.ac.uk/software/figtree/).

Mismatch distribution analysis was conducted in Arlequin 3.5 (Rogers and Harpending 1992; Harpending 1994; Excoffier et al. 2005) to test the hypothesis that spatial population expansion had occurred. The sum of squared deviations (SSD) and the raggedness index (Rag) were calculated, and significance was tested by comparing the observed and simulated mismatch distributions. If the spatial expansion model was not rejected, the expansion time (*t*) was estimated using the relationship *τ* = 2*ut* = 2*kμt*, where *μ* is the substitution rate per site per year (s/s/y) and *k* is the length of the sequence under study. An estimator of the substitution rate for angiosperm species (2.0 × 10^−9^ s/s/y) was used in this study (Wolfe et al. 1987). We also calculated Tajima's *D* in Arlequin 3.5. Tajima's *D* was used to determine whether rare mutations were significantly higher than expected (Tajima 1989), which is an indicator of recent spatial expansion (Kimura 1983).

Because no genetic variation was detected in *E. sessiliflorus* (see Results), the Mantel test, SAMOVA analysis, and mismatch analysis conducted above were applied only to *E. senticosus*.

### Ecological niche modeling

We used maximum entropy modeling (Maxent 3.3.3k) (Phillips et al. 2006) to predict the distribution of *E. senticosus* and *E. sessiliflorus* currently and during the LGM. Occurrence records were obtained from our own field sampling sites and online database, including the Chinese Virtual Herbarium (http://www.cvh.org.cn/), Global Biodiversity Information Facility (http://www.gbif.org/), and Tropicos (http://www.tropicos.org/). To mitigate potential effects of spatial sampling biases, we employed the R package spThin (Aiello‐Lammens et al. 2015) to rarefy the data to have a minimum distance of at least 10 km among them. After rarefying, 45 occurrence records of *E. senticosus* and 22 occurrence records of *E. sessiliflorus* from unique localities remained for modeling. The current and the LGM climate data were comprised of 19 bioclimatic variables from WorldClim (www.worldclim.org ) (Hijmans et al. 2005) at a resolution of 30 arc‐sec. Of the 19 climate variables, 7 variables were co‐correlated below a 0.80 Pearson |*r*| correlation and were subsequently used for species distribution modeling. These 7 bioclimatic variables were (1) mean diurnal temperature range, (2) temperature seasonality, (3) maximum temperature of the warmest month, (4) minimum temperature of the coldest month, (5) annual precipitation, (6) precipitation of the driest month, and (7) precipitation seasonality.

For LGM prediction, we used climate data from the Model for Interdisciplinary Research on Climate (MIROC), provided by the Paleoclimate Modelling Intercomparison Project (http://pmip2.lsce.ipsl.fr/). Paleocoast lines and the paleoclimate surfaces of the exposed seafloor area (assuming a sea level depression of 130 m) were estimated using seafloor topography data (ETOPO1) produced by the National Geophysical Data Center of the National Oceanic and Atmospheric Administration (Boulder, CO). We validated our models through choosing a cross‐validation set at 50 replicates using the mean area under the curve (AUC) of the receiver‐operating characteristics (ROC).

## Results

### Chloroplast genetic diversity

The three sequenced chloroplast fragments of *E. senticosus* and *E. sessiliflorus* were aligned with a consensus length of 2666 bp. Among the sampled individuals of *E. senticosus*, 17 substitutions and 3 indels were recovered and consisted of 15 haplotypes (Fig. 2A; Table S1 in the Supporting Information). However, only one haplotype was found among all sampled individuals of *E. sessiliflorus* (Fig. S1). The only haplotype (H16) of *E. sessiliflorus* was at least 17 mutational steps from those of *E. senticosus* (Fig. 2B).

In *E. senticosus*, all 15 of the haplotypes existed in NEC, and only 5 haplotypes (H1, H3, H5, H6, and H9) were shared in NC. Among the NEC populations, private haplotypes occurred in populations BS (H8 and H10), DS (H11), and CH (H14) in the Changbai Mountains and KDQ (H15) in the Xiaoxing'an Range. Most populations harbored chloroplast polymorphisms, except for 3 populations, including SHB in the Yanshan Mountains, PQG in the Taihang Mountains, and QS in the Changbai Mountains, which were fixed with single haplotypes.

**Figure 3:**
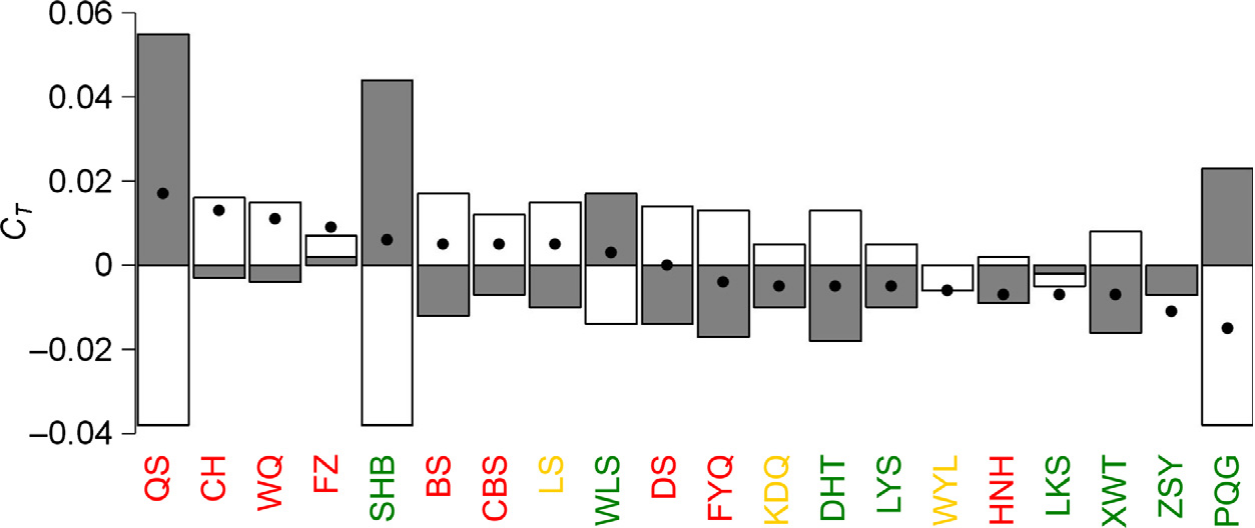
The contribution of each population in *Eleutherococcus senticosus* to the total gene diversity. The black dots on the bars represent the total gene diversity of that population (*C*_T_). Gray bars indicate the contribution to interpopulation diversity. White bars indicate the contribution to intrapopulation diversity. Sample locations from different regions were colored green for North China (NC), red for the Changbai Mountains and yellow for the Xiaoxing'an Range. The latter two areas are located in Northeast China (NEC).

High genetic diversity was found in *E. senticosus*, with *Hd* = 0.835, *π* = 0.00086, and *θ* = 0.00110. The average allele richness within populations (*R*_S_) was 2.413, and the total allele richness (*R*_T_) was 3.148 when the rarefaction size was set to 4. Population BS in the Changbai Mountains had the highest levels of haplotype diversity (*R*_S_ = 2.367 and *Hd* = 0.889), with nucleotide diversity *π* = 0.00124 and *θ* = 0.00146. Population CH in the Changbai Mountains had the most abundant haplotypes (7 haplotypes: H1, H2, H3, H7, H9, H12, and H14), with *R*_S_ = 2.269, *Hd* = 0.867, *π* = 0.00100, and *θ* = 0.00081 (Table 1). Populations QS, CH, and WQ (all in the Changbai Mountains) had the highest contributions to the total gene diversity of the species, with *C*_T_ values of 0.017, 0.013, and 0.011, respectively (Fig. 3). However, the contributions of the different populations to total gene diversity had different origins. For example, the contributions of CH and WQ were primarily due to within‐population gene diversity. In contrast, among‐population genetic divergence may account for the high value of *C*_T_ in QS, which had only one haplotype: H7 (Fig. 3).

The Mantel test for isolation by distance (IBD) failed to detect a significant correlation between the genetic and geographic distance with 999 permutations (*r* = −0.072, *P* = 0.239). *Z*‐tests of (*d*_N_−*d*_S_) of each haplotype sequence pairs were not significantly different (e.g., for the sequence pair of H16 and H6, *P* = 0.533, Table S2).

### Population genetic structure and spatial distribution of haplotypes

Extremely low population genetic differentiation was found (*G*_ST_ = 0.278 and *N*_ST_ = 0.151). The SAMOVA analysis also indicated a low population genetic structure. Among the predefined number of groups (*K* = 2–8), $F_{C_{T}}$ values were low: the highest value was 0.261 when *K* = 2 (Fig. S2), with population QS as one group and the remaining populations as the other group.

The low population genetic structure may be due to the spatial distribution of haplotypes. The most common haplotype, H1, composed 17% of the total samples and 70% of the populations (14 of 20 populations). H5 was present in 30% of the individuals and 65% of the populations. Moreover, other haplotypes were also geographically widespread despite the few individuals, such as H9, with 19 individuals found in 8 populations; H3, with 16 individuals found in 7 populations; and H6, with 28 individuals found in 7 populations (Fig. 2A; Table 1).

**Figure 4:**
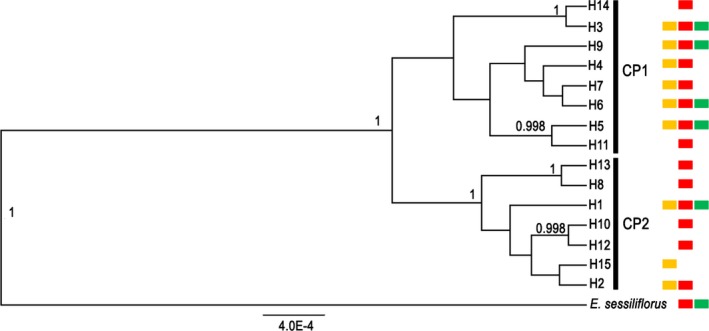
Rooted consensus trees for cpDNA haplotypes. Values on the branches are bootstrap support values for the posterior probability of the Bayesian analysis. Haplotypes distributed in different areas are marked in different colors. Colors were green for North China (NC), red for the Changbai Mountains, and yellow for the Xiaoxing'an Range.

In the rooted phylogenetic consensus tree (Fig. 4), there were two supported clades within *E. senticosus*. One clade (CP1) contained H3, H4, H5, H6, H7, H9, H11, and H14; the remaining haplotypes constituted the other clade (CP2). This tree was consistent with the haplotype network (Fig. 2B). Haplotypes distributed in the NC area were H1, H3, H5, H6, and H9, all of which were the most frequently detected haplotypes (Figs. 1A and S2).

### Historical demographic analysis

**Figure 5:**
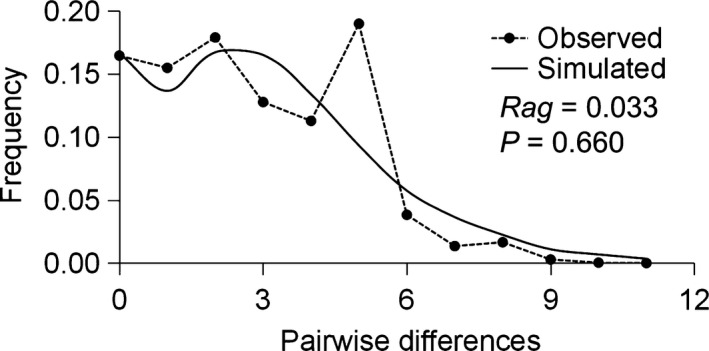
Mismatch distribution for *Eleutherococcus senticosus*. The solid line represents the simulated mismatch distribution of a stationary population. The dotted line represents the observed mismatch distribution.

Significant spatial expansion was detected for *E. senticosus* (SSD = 0.013, *P* = 0.443; Rag = 0.033, *P* = 0.660, Fig. 5). The spatial expansion parameter *τ* was 2.10 (95% HPD: 0.42–6.33). According to the relationship *τ* = 2*ut* = 2*kμt*, where *k* is 2666 bp, the expansion time (*t*) estimated for *E. senticosus* was 0.20 Mya (95% HPD: 0.04–0.59). In addition, another indicator, Tajima's *D* = −0.576 (*P* = 0.314), did not significantly deviate from a neutral pattern, suggesting that there was no significant signal of a recent spatial expansion.

### Ecological niche modeling

**Figure 6:**
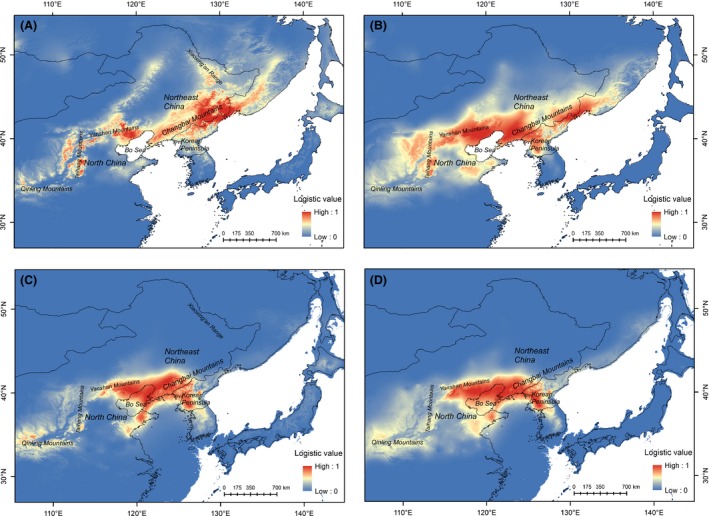
Results of the predicted distribution probability from ecological niche modeling (ENM) for current climatic conditions of *Eleutherococcus senticosus* (A) and *E. sessiliflorus* (B) and for the Last Glacial Maximum (LGM, *c*. 21,000–18,000 years before present) of *E. senticosus* (C) and *E. sessiliflorus* (D) using data from the Model for Interdisciplinary Research on Climate (MIROC).

The Maxent model performed well for both *E. senticosus* and *E. sessiliflorus*, with AUC values of 0.971 ± 0.011 and 0.983 ± 0.015 (mean ± SD), respectively. Under the present climate, the distribution of *E. senticosus* was predicted to occur in the NEC region, the NC region, and the Korean Peninsula with high probability (Fig. 6A). *E. sessiliflorus* was predicted to occupy nearly all of the same areas as *E. senticosus*, except for the northernmost area (e.g., the Xiaoxing'an Range) in NEC (Fig. 6B). The predicted distribution was generally consistent with the actual distribution of each species (Fig. 2A).

Under the LGM climate, the probability of each species being distributed in the northern and eastern margins of NEC decreased, whereas the probability of each species being distributed in southern NEC and northern NC as well as in the Qinling Mountains in the south increased (Fig. 6C and D). The difference in the predicted distribution between the two species was relatively minor.

## Discussion

Although *E. senticosus* and *E. sessiliflorus* are two sympatric species of the genus *Eleutherococcus*, their genetic patterns were quite different. A high genetic diversity was observed for *E. senticosus*, with 15 haplotypes detected from 2666 bp cpDNA. In contrast, no genetic diversity was detected in *E. sessiliflorus* (only one haplotype, H16) (Fig. 2B). It might be normal that different species within the same region were detected different counts of chloroplast haplotypes, such as *Acer mono* (10 haplotypes from 2140 bp sampled fragments) (Guo et al. 2014), *Quercus mongolica* (9 haplotypes from 3979 bp) (Zeng et al. 2015), *Ostryopsis davidiana* (3 haplotypes from 1300 bp) (Tian et al. 2009), and *Juglans mandshurica* (2 haplotypes from 5850 bp) (Bai et al. 2010). However, some species still had obvious low genetic variation even with longer sampled fragment size, such as *Juglans mandshurica* and *E. sessiliflorus*. *E. sessiliflorus* had even lower genetic diversity than *Juglans mandshurica* because no genetic variation was detected in distant populations despite sampling more than 11,000 bp of 13 chloroplast loci during the pre‐experimental stage (see the Methods section).

In addition to the same sequencing size sampled in both *Eleutherococcus* species, all of the sampled locations of *E. sessiliflorus*, except for DQG and BSh, overlapped with those of *E. senticosus*. Both species are congeneric shrubs with similar seeds and pollen dispersal systems, generation times, and species ranges. The mating systems were both outcrossing‐dominated and self‐compatible (Liu et al. 2002b). The aforementioned factors are crucial in affecting the levels of species genetic variation (Cutter et al. 2013). Besides, with the low sample size, it is possible to miss important genetic variation (61 individuals from *E. sessiliflorus*, compared to 185 from *E. senticosus*). However, the 61 individuals from 9 populations of *E. sessiliflorus* are scattered across the species range. The scattered sample strategy should have characterized the important genetic variation of *E. sessiliflorus*. We propose that two possible scenarios may have generated the extremely low genetic variation in *E. sessiliflorus*: (1) a severe bottleneck, which resulted in a low extant effective population size (Charlesworth et al. 2003; Leffler et al. 2012); and (2) selective sweeps, which may have eliminated standing genetic variation (Charlesworth et al. 2003; Frankham 2012). Chloroplast is usually considered as neutral, but one or more trans‐species selective sweeps of the chloroplasts have been shown to occur in many *Salix* species, resulting in an unusual shared pattern of chloroplast haplotypes (Percy et al. 2014). However, our study suggested that the second scenario was less likely in *E. sessiliflorus*, because *Z*‐tests of (*d*_N_−*d*_S_) in *matK* were not significant and thus do not permit the rejection of the hypothesis that these sequences have evolved neutrally.

A severe bottleneck may have occurred during the Quaternary climatic oscillation, when harsh environments during the glaciation may have eliminated populations in the northern range. For example, a study by Jakob et al. (2007) has indicated that the lack of cpDNA variation in grass species *Hordeum gussoneanum*, based on both DNA sequencing and microsatellite length variation, might be due to a severe and relatively recent bottleneck that erased all of the chloroplast variation. Although the total absence of genetic variation throughout the range of each species has not been commonly found in publications, extremely low regional genetic variation, especially in the northern portion of the range of a species, is often observed in temperate species (Palmé and Vendramin 2002; Grivet and Petit 2003; Dorken and Barrett 2004; Magni et al. 2005; Bai et al. 2010, 2015; Sakaguchi et al. 2012; Guo et al. 2014). The regional and simultaneous absence of genetic variation in the range of northern species provides evidence that many of these northern populations may have experienced a recent severe bottleneck due to the LGM (Brochmann et al. 2003; Hewitt 2004, 2011; Soltis et al. 2006). As *E. sessiliflorus* distributes in lower altitude than *E. senticosus*, it is possible that the former species is cold‐intolerant compared to the latter one. Thus, during the glacial period, *E. sessiliflorus* may have experienced much more extent of contraction than *E. senticosus*. However, ecological experiments comparing the cold tolerances of the two species should be taken in the future to gain more solid proofs.

In contrast to *E. sessiliflorus*,* E. senticosus* has relatively high genetic diversity. However, low genetic differentiation and no phylogeographic structure were recovered throughout the range of species, because no clear spatial genetic group could be divided based on SAMOVA analysis, and the *G*_ST_ and/or $F_{C_{T}}$ values were considerably lower than those of other temperate species, which could be higher than 0.8 (Petit et al. 2003; Ye et al. 2015). In addition, no IBD pattern was found. And the signal of spatial expansion was not explicit, as mismatch analysis indicated an old expansion (0.20 Mya, 0.04–0.59), while Tajima's *D* revealed no signal of expansion. Nonetheless, all the analyses supported that no expansion started after LGM. Collectively, we infer that multiple LGM refugia may have contributed to the high genetic diversity and low genetic differentiation pattern of *E. senticosus*.

The genetic diversity in the NEC region was generally higher than that in the NC region. Most populations in NC had only 1–2 haplotypes, with a maximum of 4, whereas populations in NEC generally had 3–7 haplotypes. The average rarified allelic richness in NC (*R*_S_ = 0.973) was lower than that in NEC (*R*_S_ = 1.707), although this difference was not significant (*P* = 0.405). The *C*_T_ value in NC was negative, except for populations SHB and WLS, whereas 6 populations in the Changbai Mountains had positive values (Fig. 3), which indicated that these populations differentially contributed to overall species genetic diversity. In addition, 4 private haplotypes were detected in the Changbai Mountains, but no haplotypes were detected in the NC region. Two scenarios may explain the findings in the Changbai Mountains. First, the Changbai Mountains may have acted as LGM refugia. Long‐term survivorship of refugium populations may result in higher genetic diversity and private haplotypes sustained compared to other newly colonized populations (Hewitt 1996, 2000; Petit et al. 2003). The Changbai Mountains have been suggested to be LGM refugia for several studied temperate forest species (Bai et al. 2010, 2015; Guo et al. 2014; Bao et al. 2015; Zeng et al. 2015). Second, the Changbai Mountains have acted as a recent admixture of multiple LGM refugia. Petit et al. (2003) have indicated that the highest genetic diversity of several European forest species occurs at relatively high latitudes, which has been suggested to be a consequence of lineage admixture from 3 independent southern refugia. For *E. senticosus*, we propose that both of the above‐suggested scenarios may have occurred in the Changbai Mountains.

Although NC had low genetic diversity and no private haplotypes, we still could not rule out the possibility of NC as a LGM refugium, due to its special geographic location. NC has been considered an important refugium and source of postglacial expansion to the NEC region for many studied temperate forest species (Hu et al. 2008; Bai et al. 2010, 2015; Guo et al. 2014; Zeng et al. 2015). For *E. senticosus*, if the Changbai Mountains have LGM refugia (discussed above), NC should have a higher potential to sustain LGM populations because NC is located in the South and should therefore have more suitable habitats (Fig. 6C). The NEC region could receive migrants from NC due to northward postglacial expansion, which could elevate the genetic variation in NEC and lead to genetic variation sharing between NEC and NC. Hence, a relatively high genetic diversity in the NEC region could also be a result of lineage admixture from separate refugia including NC.

In our study, some populations in the Xiaoxing'an Range had relatively high levels of genetic diversity, such as population LS. Moreover, the northernmost population KDQ harbored a private haplotype: H15 (Fig. 2A). Cryptic refugia of *E. senticosus* probably existed in the Xiaoxing'an Range, as suggested by other codistributed species (Hu et al. 2008; Bao et al. 2015; Zeng et al. 2015). However, the lack of fossil and geological evidence makes this a weak inference (Qiu et al. 2011; Tzedakis et al. 2013; Cao et al. 2015). As the nuclear genome has a higher genetic diversity than chloroplast, it can provide more resolution in future studies to understand the possibility of northern cryptic refugia.

Although the two codistributed *Eleutherococcus* species had contrasting genetic patterns, our ENM results did not show big differences between them. ENM suggested that both species could sustain populations in NC, south of NEC, and the Korean Peninsula, as well as the Bo Sea, during the LGM (Fig. 6C and D). In recent years, ENM is generally suggested as a standard analysis for phylogeography studies, because the approach provides independent clues for inferring suitable habitats for species during the glacial period. However, an obvious flaw may make the inference unreliable. Firstly, the current niche modeling approach considers only climate variables and does not account for biological interactions, which can also be critical to species survival in a community (Ricklefs 1987; Hooper et al. 2005). Such data are largely based on ecological surveys, which vary from community to community. However, data of this type are not often collected (Martin‐Albarracin et al. 2015; Valladares et al. 2015). Thus, species may fail to survive in the predicted, but climatically suitable habitat. Secondly, coarse resolution of the ENM can smooth the differences between microhabitats. Hence, it is hard to find cryptic refugia as well as microhabitats differences between codistributed species (Dellicour et al. 2014, 2015). Although habitats of the two *Eleutherococcus* species overlap at a regional scale, the small‐scale niche differences (e.g., altitude) are ignored by the current simulations due to the low resolution of ancient climate data. Hence, we include ENM results in our study, but considering the low resolution and significant flaws, we do not think it provides reliable supporting information for understanding the contrasting genetic patterns between the two codistributed *Eleutherococcus* species.

## Conclusions and Implications

Although *E. senticosus* and *E. sessiliflorus* are closely related and overlap in their ranges, contrasting genetic patterns were detected based on cpDNA sequencing and coherent sampling strategies of these two congeneric species. *E. sessiliflorus* had no genetic variation across all of the sampled loci, whereas *E. senticosus* had considerably higher levels of genetic diversity. We infer that a recent severe bottleneck likely occurred in *E. sessiliflorus*. For *E. senticosus*, relatively higher genetic diversity was found in the Changbai Mountains than in NC. We suggest that both NC and the Changbai Mountains may have sustained LGM refugia. Lineage admixture from separate LGM refugia may have contributed to the high genetic variation in the Changbai Mountains. There were signals indicating that the Xiaoxing'an Range may have had cryptic LGM refugia. Confirmation of this possibility will require more evidence. Large amounts of nuclear genetic markers will be needed to accurately infer the origin of the contrasting genetic patterns between these codistributed and closely related species.

The Korean Peninsula and the Bo Sea are other sources of potential LGM refugia for *E. senticosus*, as predicted by ENM. The Bo Sea was converted to land during the LGM due to the lowering of the sea level. Analysis of samples from the Korean Peninsula would be beneficial for inferring demographic history in future studies. Currently, however, we believe that the unsampled status of the Korean Peninsula and the Bo Sea will not diminish the importance of the conclusions that are made in this paper. Both the Korean Peninsula and Bo Sea refugia may contribute to the extant local gene pool of the Changbai Mountains. Some private haplotypes in the Changbai Mountains may have originated from the two above unsampled LGM refugia. However, no IBD pattern was recovered. Populations with high genetic diversity were located at medium to high latitudes (such as WQ, CH, and LS). This location is considerably further north than would have been possible to admix in the region containing the three potential southern refugia (NC, the Bo Sea, and the Korean Peninsula, Fig. 2). Hence, the Changbai Mountains may have sustained separate refugia during the LGM.

## Conflict of Interest

None declared.

## Supporting information


**Figure S1.** Geographic distribution of chloroplast haplotype in *Eleutherococcus sessiliflorus*.
**Figure S2.** SAMOVA of *E. senticosus* based on cpDNA.
**Table S1.** Designation of the cpDNA haplotypes detected at the 3 chloroplast loci among 20 *E. senticosus* populations and 9 *E. sessiliflorus* populations
**Table S2.** *Z*‐tests of (*d*_N_–*d*_S_) of each haplotype sequence pairs
