# Supplementary material for: Contrasting genetic patterns between two coexisting Eleutherococcus species in northern China
Source: Ecol Evol. 2016 Apr 12;6(10):3311–24. doi: 10.1002/ece3.2118 (PMC4833501; doi:10.1002/ece3.2118)
Supplement: Supplementary file 1 — Figure S1. Geographic distribution of chloroplast haplotype in Eleutherococcus sessiliflorus. Figure S2. SAMOVA of E. senticosus based on cpDNA. Table S1. Designation of the cpDNA haplotypes detected at the 3 chloroplast loci among 20 E. senticosus populations and 9 E. sessiliflorus populations Table S2. Z‐tests of (d N–d S) of each haplotype sequence pairs [file ECE3-6-3311-s001.doc]

**Supporting Information Figs. S1-S2 and Tables S1-S2**

**Figure S1.** Geographic distribution of chloroplast haplotype in *E. sessiliflorus*. The size of the pies is proportional to the sample size of each population.


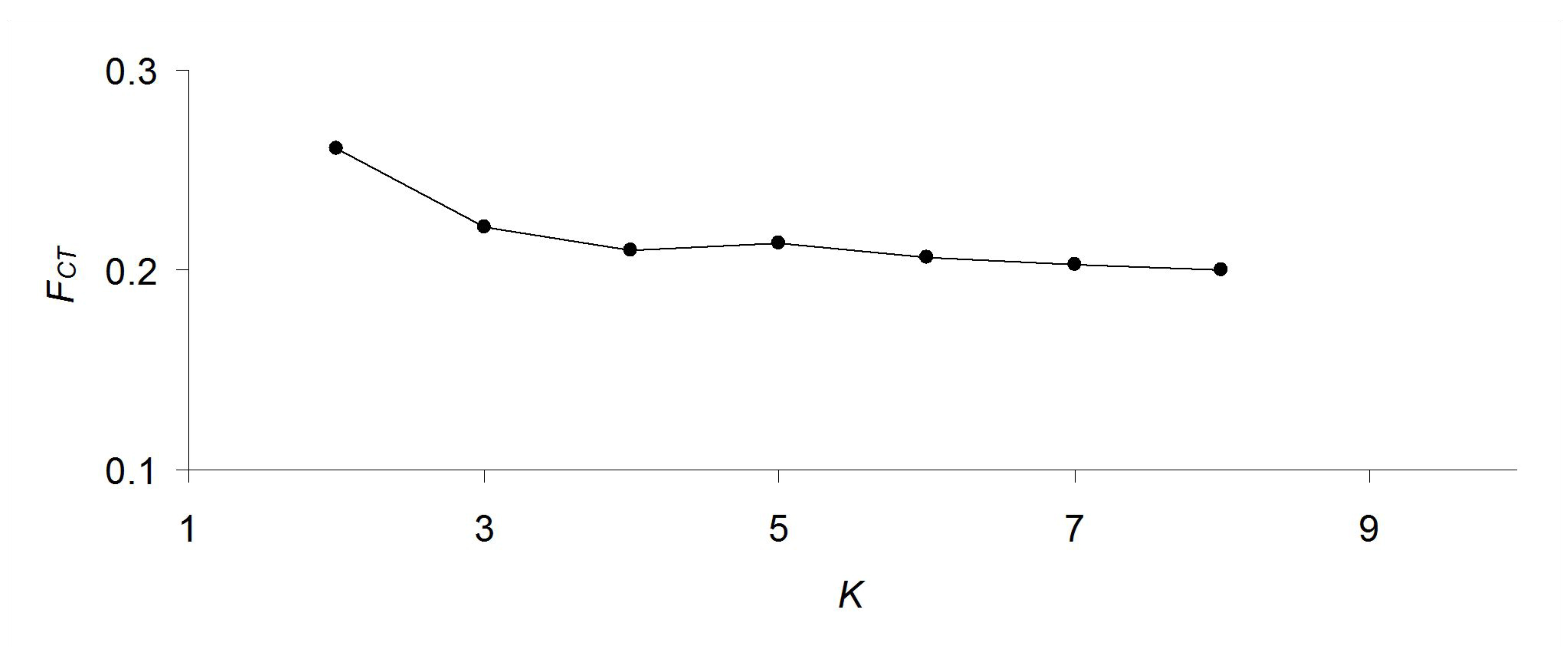


**Figure S2.** SAMOVA of *E. senticosus* based on cpDNA. The solid line represents the change in variance among groups (*F_CT_*) with group numbers (*K*) defined from 2 to 8.

| **Table S1.** Designation of the cpDNA haplotypes detected at the three chloroplast loci among 20 *E. senticosus* populations and 9 *E. sessiliflorus* populations | | | | | | | | | | | | | | | | | | | | | | | | | | | | | | | | | | | | | | | | |
| --- | --- | --- | --- | --- | --- | --- | --- | --- | --- | --- | --- | --- | --- | --- | --- | --- | --- | --- | --- | --- | --- | --- | --- | --- | --- | --- | --- | --- | --- | --- | --- | --- | --- | --- | --- | --- | --- | --- | --- | --- |
| Haplotype | n | Location of polymorphism sites | | | | | | | | | | | | | | | | | | | | | | | | | | | | | | | | | | | | | | |
|  |  | *trnL*-*trnF* | | | | | | |  | *rpl16* | | | | | | | | | | |  | *matK* | | | | | | | | | | | | | | | | | | |
|  |  | 65 | 162 | 166 | 550 | 699 | 770 | 814 |  | 948 | 972 | 974 | 1155 | 1168 | 1198 | 1219 | 1380 | 1396 | 1440 | 1583 |  | 1802 | 1818 | 1830 | 1911 | 1919 | 2014 | 2080 | 2151 | 2238 | 2289 | 2317 | 2357 | 2372 | 2431 | 2541 | 2552 | 2570 | 2621 | 2630 |
| H1 | 31 | C | - | C | I^9^ | C | C | I^18^ |  | T | A | - | G | G | G | T | C | A | G | A |  | C | C | T | T | A | A | C | T | C | T | C | G | T | C | A | C | G | T | T |
| H2 | 4 | C | - | C | - | C | C | I^18^ |  | T | A | I^6^ | G | G | G | T | T | A | G | A |  | C | C | T | T | A | A | C | T | C | T | C | G | T | C | A | C | G | T | T |
| H3 | 16 | C | I^2^ | C | - | C | C | I^18^ |  | T | A | - | G | G | G | T | T | A | G | A |  | C | C | T | T | A | A | C | T | C | C | C | G | C | C | A | C | G | T | G |
| H4 | 3 | C | - | C | - | C | C | I^18^ |  | T | A | - | G | G | G | T | T | A | G | A |  | C | C | T | C | A | A | C | T | C | C | C | G | C | C | A | C | G | T | T |
| H5 | 56 | C | - | C | - | C | C | I^18^ |  | T | A | - | G | G | G | T | T | A | G | A |  | C | C | G | T | A | A | C | T | C | C | C | G | C | C | A | C | G | T | T |
| H6 | 28 | C | - | C | - | C | C | I^18^ |  | T | A | - | G | G | G | T | T | A | G | A |  | C | C | T | T | A | A | C | T | C | C | C | G | C | C | A | C | G | T | T |
| H7 | 15 | C | - | C | - | C | C | I^18^ |  | T | A | - | G | G | G | T | T | A | G | A |  | C | G | T | T | A | A | C | T | C | C | C | G | C | C | A | C | G | T | T |
| H8 | 1 | C | - | C | - | C | T | I^18^ |  | T | A | - | G | G | G | T | T | A | G | A |  | C | C | T | T | A | A | C | T | C | T | C | G | T | C | T | T | G | T | T |
| H9 | 19 | C | - | C | - | C | C | I^18^ |  | T | A | - | G | G | A | T | T | A | G | A |  | C | C | T | T | A | A | C | T | C | C | C | G | C | C | A | C | G | T | T |
| H10 | 1 | C | - | C | - | C | C | I^18^ |  | T | A | - | G | G | G | T | T | A | G | A |  | C | C | T | T | A | A | C | T | T | T | T | G | T | C | A | C | G | T | T |
| H11 | 1 | C | - | C | - | C | C | I^18^ |  | T | A | - | G | G | G | T | T | A | A | A |  | C | C | G | T | A | C | C | T | C | C | C | G | C | C | A | C | G | T | T |
| H12 | 5 | C | - | C | - | C | C | I^18^ |  | T | A | - | G | G | G | T | T | A | G | A |  | C | C | T | T | A | A | C | T | C | T | T | G | T | C | A | C | G | T | T |
| H13 | 3 | C | - | C | - | C | T | I^18^ |  | G | A | - | G | G | G | T | T | A | G | A |  | C | C | T | T | A | A | T | T | C | T | C | G | T | C | T | T | G | T | T |
| H14 | 1 | C | I^2^ | C | - | C | C | I^18^ |  | T | A | - | G | G | G | T | C | A | G | A |  | C | C | T | T | A | A | C | T | C | C | C | G | C | C | A | C | G | T | G |
| H15 | 1 | C | - | C | - | C | C | I^18^ |  | T | A | - | G | G | G | T | T | A | G | A |  | C | C | T | T | A | A | C | T | C | T | C | G | T | C | A | C | G | T | T |
| H16 | 61 | T | - | T | - | T | C | - |  | T | C | - | A | T | G | G | T | C | G | T |  | A | C | T | T | C | A | C | C | C | C | C | T | C | T | A | C | C | C | T |
| n, number of indviduals; I^x^, insertion (‘x’ is the number of bases inserted). | | | | | | | | | | | | | | | | | | | | | | | | | | | | | | | | | | | | | | | | |

| **Table S2.** Z-tests of (*d_N_* - *d_S_*) of each haplotype sequence pairs | | | | | | | | | | | | | | | | |
| --- | --- | --- | --- | --- | --- | --- | --- | --- | --- | --- | --- | --- | --- | --- | --- | --- |
|  | H1 | H2 | H3 | H4 | H5 | H6 | H7 | H8 | H9 | H10 | H11 | H12 | H13 | H14 | H15 | H16 |
| H1 |  | 0.000 | -0.388 | -1.253 | -1.247 | -0.715 | -0.393 | -0.692 | -0.715 | -1.459 | -0.997 | -1.035 | -1.234 | -0.388 | 0.000 | 0.046 |
| H2 | 1.000 |  | -0.388 | -1.253 | -1.247 | -0.715 | -0.393 | -0.692 | -0.715 | -1.459 | -0.997 | -1.035 | -1.234 | -0.388 | 0.000 | 0.046 |
| H3 | 0.699 | 0.699 |  | -0.698 | -0.668 | 0.971 | 1.390 | -0.738 | 0.971 | -1.330 | -0.371 | -0.923 | -1.184 | 0.000 | -0.388 | 0.838 |
| H4 | 0.213 | 0.213 | 0.486 |  | -1.447 | -1.035 | -0.705 | -1.400 | -1.035 | -1.820 | -1.197 | -1.531 | -1.704 | -0.698 | -1.253 | -0.140 |
| H5 | 0.215 | 0.215 | 0.506 | 0.150 |  | -0.991 | -0.672 | -1.393 | -0.991 | -1.891 | 1.040 | -1.568 | -1.712 | -0.668 | -1.247 | -0.135 |
| H6 | 0.476 | 0.476 | 0.333 | 0.303 | 0.324 |  | 1.032 | -0.973 | 0.000 | -1.515 | -0.667 | -1.156 | -1.380 | 0.971 | -0.715 | 0.625 |
| H7 | 0.695 | 0.695 | 0.167 | 0.482 | 0.503 | 0.304 |  | -0.749 | 1.032 | -1.314 | -0.371 | -0.929 | -1.181 | 1.390 | -0.393 | 0.836 |
| H8 | 0.490 | 0.490 | 0.462 | 0.164 | 0.166 | 0.332 | 0.455 |  | -0.973 | -1.600 | -1.196 | -1.215 | -1.066 | -0.738 | -0.692 | -0.329 |
| H9 | 0.476 | 0.476 | 0.333 | 0.303 | 0.324 | 1.000 | 0.304 | 0.332 |  | -1.515 | -0.667 | -1.156 | -1.380 | 0.971 | -0.715 | 0.625 |
| H10 | 0.147 | 0.147 | 0.186 | 0.071 | 0.061 | 0.132 | 0.191 | 0.112 | 0.132 |  | -1.699 | -1.069 | -1.891 | -1.330 | -1.459 | -0.902 |
| H11 | 0.321 | 0.321 | 0.711 | 0.233 | 0.301 | 0.506 | 0.711 | 0.234 | 0.506 | 0.092 |  | -1.362 | -1.534 | -0.371 | -0.997 | 0.047 |
| H12 | 0.303 | 0.303 | 0.358 | 0.128 | 0.119 | 0.250 | 0.355 | 0.227 | 0.250 | 0.287 | 0.176 |  | -1.605 | -0.923 | -1.035 | -0.482 |
| H13 | 0.220 | 0.220 | 0.239 | 0.091 | 0.089 | 0.170 | 0.240 | 0.288 | 0.170 | 0.061 | 0.128 | 0.111 |  | -1.184 | -1.234 | -0.753 |
| H14 | 0.699 | 0.699 | 1.000 | 0.486 | 0.506 | 0.333 | 0.167 | 0.462 | 0.333 | 0.186 | 0.711 | 0.358 | 0.239 |  | -0.388 | 0.838 |
| H15 | 1.000 | 1.000 | 0.699 | 0.213 | 0.215 | 0.476 | 0.695 | 0.490 | 0.476 | 0.147 | 0.321 | 0.303 | 0.220 | 0.699 |  | 0.046 |
| H16 | 0.964 | 0.964 | 0.404 | 0.889 | 0.893 | 0.533 | 0.405 | 0.743 | 0.533 | 0.369 | 0.962 | 0.630 | 0.453 | 0.404 | 0.964 |  |
| Probability (black), probability computed (< 0.05 for hypothesis rejection at the 5% level); Statistic (blue), statistic used to compute the probability. | | | | | | | | | | | | | | | | |
